# Aging beyond menopause selectively decreases CD8+ T cell numbers but enhances cytotoxic activity in the human endometrium

**DOI:** 10.1186/s12979-022-00312-w

**Published:** 2022-11-12

**Authors:** Zheng Shen, Mickey V. Patel, Marta Rodriguez-Garcia, Charles R. Wira

**Affiliations:** 1 Department of Microbiology and Immunology, Geisel School of Medicine at Dartmouth, One Medical Center Drive, Lebanon, NH 03756 USA; 2 Department of Immunology, Tufts University School of Medicine, Boston, MA USA

**Keywords:** Aging, Menopause, Endometrium, CD8+ T cells, Cytotoxic activity

## Abstract

**Background:**

Regulation of endometrial (EM) CD8+ T cells, which provide protection through cell-mediated cytotoxicity, is essential for successful reproduction, and protection against sexually transmitted infections and potential tumors. We have previously demonstrated that EM CD8+ T cell cytotoxicity is suppressed directly and indirectly by sex hormones and enhanced after menopause. What remains unclear is whether CD8+ T cell protection and the contribution of tissue-resident (CD103+) and non-resident (CD103-) T cell populations in the EM change as women age following menopause.

**Results:**

Using hysterectomy EM tissues, we found that EM CD8+ T cell numbers declined significantly in the years following menopause. Despite an overall decline in CD8+ T cells, cytotoxic activity per cell for both CD103- and CD103 + CD8+ T cells increased with age. Investigation of the underlying mechanisms responsible for cytotoxicity indicated that the percentage of total granzyme A and granzyme B positive CD8+ T cells, but not perforin, increased significantly after menopause and remained high and constant as women aged. Additionally, baseline TNFα production by EM CD8+ T cells increased significantly in the years following menopause, and estradiol suppressed TNFα secretion. Moreover, in response to PMA activation, TNFα and IFNγ were significantly up-regulated, and CD103-CD8+ T cells up-regulation of TNFα, IFNγ and IL-6 increased as women aged.

**Conclusions:**

Understanding the underlying factors involved in regulating cell-mediated protection of the EM by CD8+ T cells will contribute to the foundation of information essential for developing therapeutic tools to protect women against gynecological cancers and infections as they age.

**Supplementary Information:**

The online version contains supplementary material available at 10.1186/s12979-022-00312-w.

## Background

Unique among mucosal sites, the immune system in the human uterine endometrium (EM) has evolved to create a cyclic permissive environment conducive to successful reproduction, while maintaining a level of protection against incoming pathogens and potential tumors. To accomplish this, the immune system in the human female reproductive tract (FRT) is regulated by sex hormones (estradiol and progesterone) in pre-menopausal women [1]. With menopause, and due to the cessation of ovarian hormone production, the immune environment responds with profound changes [2, 3]. Since the average age at menopause is 52 years in the United States [4], and the average life expectancy of women is 81 years [5], this results in women having a uniquely long survival potential in a post-menopausal environment characterized by low concentrations of sex hormones [6].

As women age in the decades following menopause, their morbidity and mortality is significantly affected by the increased incidence in gynecological cancers and genitourinary infections. This represents a growing public health problem, recognizing the rapid expansion of the aging population globally, and that women represent two thirds of this population [7–10].

Central to adaptive immune protection in the FRT are the T cells which are a major constituent of leukocytes (30–60%) of which two-thirds of these are CD8+ T cells in the human EM [11]. CD8+ T cells in the EM can be divided in tissue-resident (CD103+) and non-resident (CD103-) populations. After menopause, when reproductive function is lost, the CD8+ T cells in the EM undergo changes in distribution and function [3, 12–14]. We have previously shown that the percentage of total CD8+ T cells and CD103 + CD8+ T cells increase in the EM following menopause [13]. Cytotoxic activity of EM CD8+ T cells, measured as direct or indirect killing of allogeneic target cells, is significantly suppressed in pre-menopausal women compared to post-menopausal women [12, 14]. Within pre-menopausal women, cytotoxic activity is further suppressed during the secretory phase of the menstrual cycle, when implantation and pregnancy are likely to occur in the EM. Similar to functional regulation, the distribution of CD8+ T cells changes across the menstrual cycle and with menopause. For example, during the secretory phase, EM CD8+ T cells form part of large clusters of lymphoid aggregates that dispersed following menstruation and are absent after menopause [15, 16].

We have recently reported multiple mechanisms that suppress CD8+ T cell cytotoxic activity before and after menopause, including direct and indirect effects of sex hormones and transforming growth factor beta (TGFβ) [14, 17]. However, the suppressive effects of TGFβ decreased after menopause [14]. The menopausal transition also has other potent effects of CD8+ T cell function, including increased degranulation capacity and changes in the composition of granzyme proteins, with preferential production of granzyme A (GZMA) compared to granzyme B (GZMB) following stimulation with phorbol 12- myristate 13-acetate (PMA)/ionomycin [14]. Interestingly, while CD103+ and CD103-CD8+ T cells have differential cytotoxic capacity, both populations equally undergo changes in their granzyme profiles and increase degranulation after menopause. However, the post-menopausal period in women can last for several decades and it is unknown what further changes may occur with increasing age after menopause.

Here, using endometrial samples from women ranging from 26 to 81 years of age, we investigated the changes in distribution and function that occur in EM CD8+ T cells with aging. We found that aging following menopause leads to profound changes in EM CD8+ T cell numbers, cytotoxic activity, granular content, and the production of cytokines and chemokines by both resident and non-resident EM CD8+ T cells. Understanding the underlying factors involved in regulating cell-mediated protection by CD8+ T cells in the EM will contribute to the foundation of information essential for developing therapeutic tools to protect women against gynecological cancers and infections as they age.

## Results

### CD8+ T cell numbers decrease with age in the endometrium

**Fig. 1:**
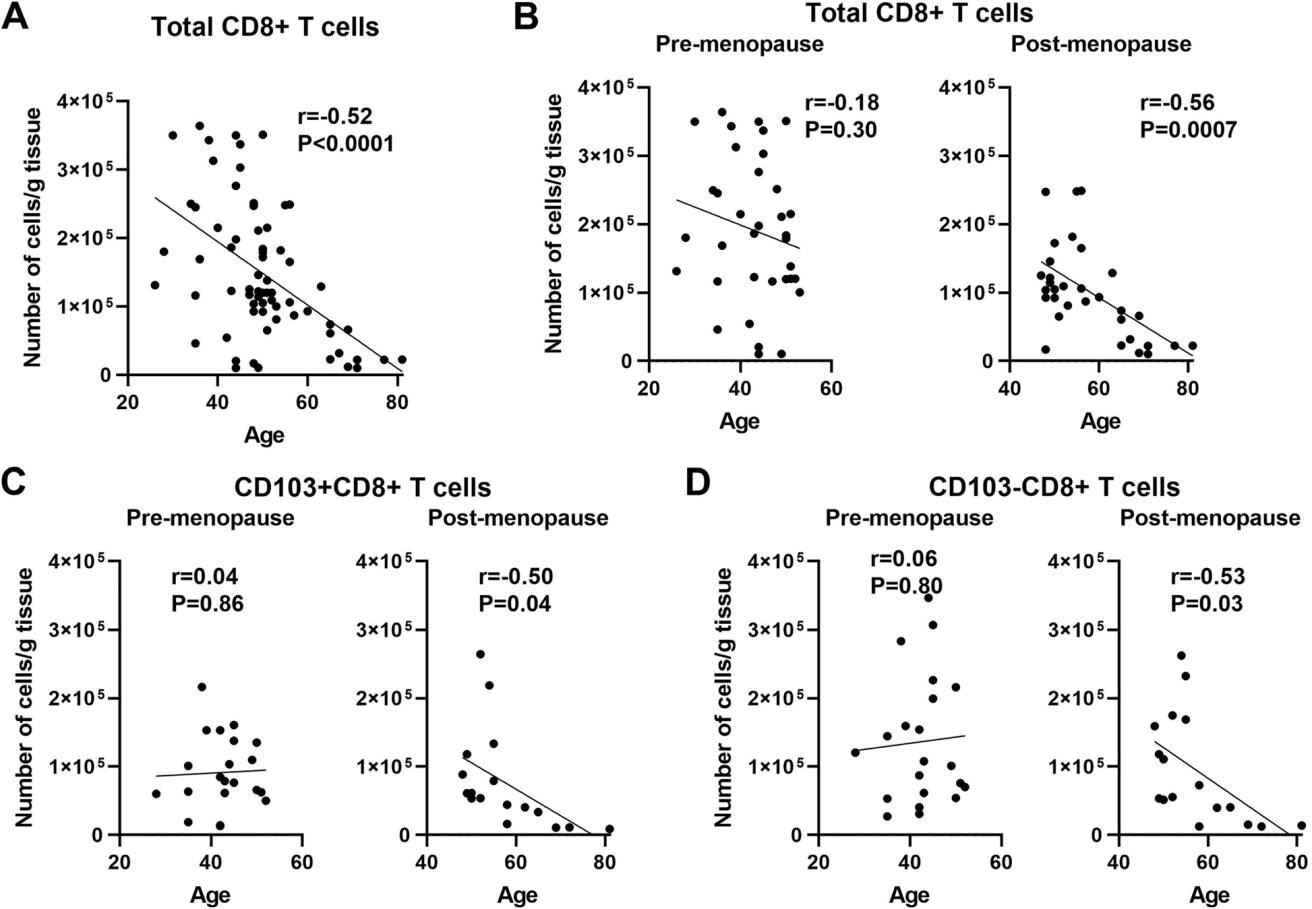
CD8+ T cell numbers decrease with age in the EM. **A** Correlation between number of CD8+ T cells recovered per gram of EM tissue after magnetic bead isolation and age from the entire study population (*n* = 68). **B** Correlation between number of CD8+ T cells recovered per gram of EM tissue after separating pre- (left; *n* = 35) and post-menopausal cells (right; *n* = 33) women. **C** Correlation between number of CD103 + CD8+ T cells recovered per gram of EM tissue and age from pre- (left; *n* = 21) or post-menopausal (right; *n* = 17) women. **D** Correlation between number of CD103-CD8+ T cells recovered per gram of EM tissue and age from pre- (left; *n* = 21) or post-menopausal (right; *n* = 17) women. Each dot represents a single patient. Spearman test

Previous studies from our laboratory have demonstrated that EM CD8+ T cell cytotoxicity varies with the stage of the menstrual cycle, is markedly increased following menopause, and is suppressed both directly and indirectly by sex hormones [12, 14, 17]. In the present study, we investigated the underlying mechanisms that influence CD8+ T protection in the EM as women age following menopause. First, we measured the number of EM CD8+ T cells per gram of tissue in each patient. Consistent with our past findings [11], the number of EM CD8+ T cells per gram of tissue was significantly lower in post-menopausal versus pre-menopausal women (Supplementary Fig. 1A). In both populations there was a wide range of total numbers of EM CD8+ T cells, ranging from 1.02 × 10^4^ to 3.64 × 10^5^ cells/g and 1 × 10^4^ to 2.49 × 10^5^ cells/g in pre- and post-menopausal women respectively. We then stratified CD8+ T cell numbers as a function of age across the entire study population. As seen in Fig. 1A, we found that EM CD8+ T cell numbers decreased significantly with increasing age in the entire population. However, this overall decrease in CD8+ T cell number masked two distinct profiles in the pre- and post-menopausal populations. In pre-menopausal women, the number of EM CD8+ T cells/g did not significantly change with increasing age prior to menopause (range 26–53 years; *n* = 35) (Fig. 1B, left). In contrast, in post-menopausal women EM CD8+ T cell numbers declined significantly with increasing age (range 48–81 years; *n* = 33) (Fig. 1B, right).

In previous studies, we found that EM CD8+ T cells consist of both tissue resident cells and non-resident CD8+ T cells that are CD103+ and CD103-, respectively [13, 14]. To determine the extent to which age influences their presence in the EM, CD8+ T cells were divided into CD103+ and CD103- cells. As seen in Supplementary Fig. 1B, the number of CD103+ (left) or CD103- (right) CD8+ T cells per gram of tissue indicated no differences between pre- and post-menopausal women. As a percentage of total CD8+ T cells, the percent of CD103+ from pre- and post-menopause were 41 and 49%, respectively. However, when stratified by age, as shown in Fig. 1C and D, both CD103+ and CD103-CD8+ T cell numbers decreased significantly with increasing age in post-menopausal women (range 49–81 years; *n* = 17). In contrast, there were no differences in CD103+ and CD103-CD8+ T cell numbers with increasing age in pre-menopausal women (range 28–51 years; *n* = 21).

### CD8+ T cells cytotoxic activity increases with age in the endometrium

**Fig. 2:**
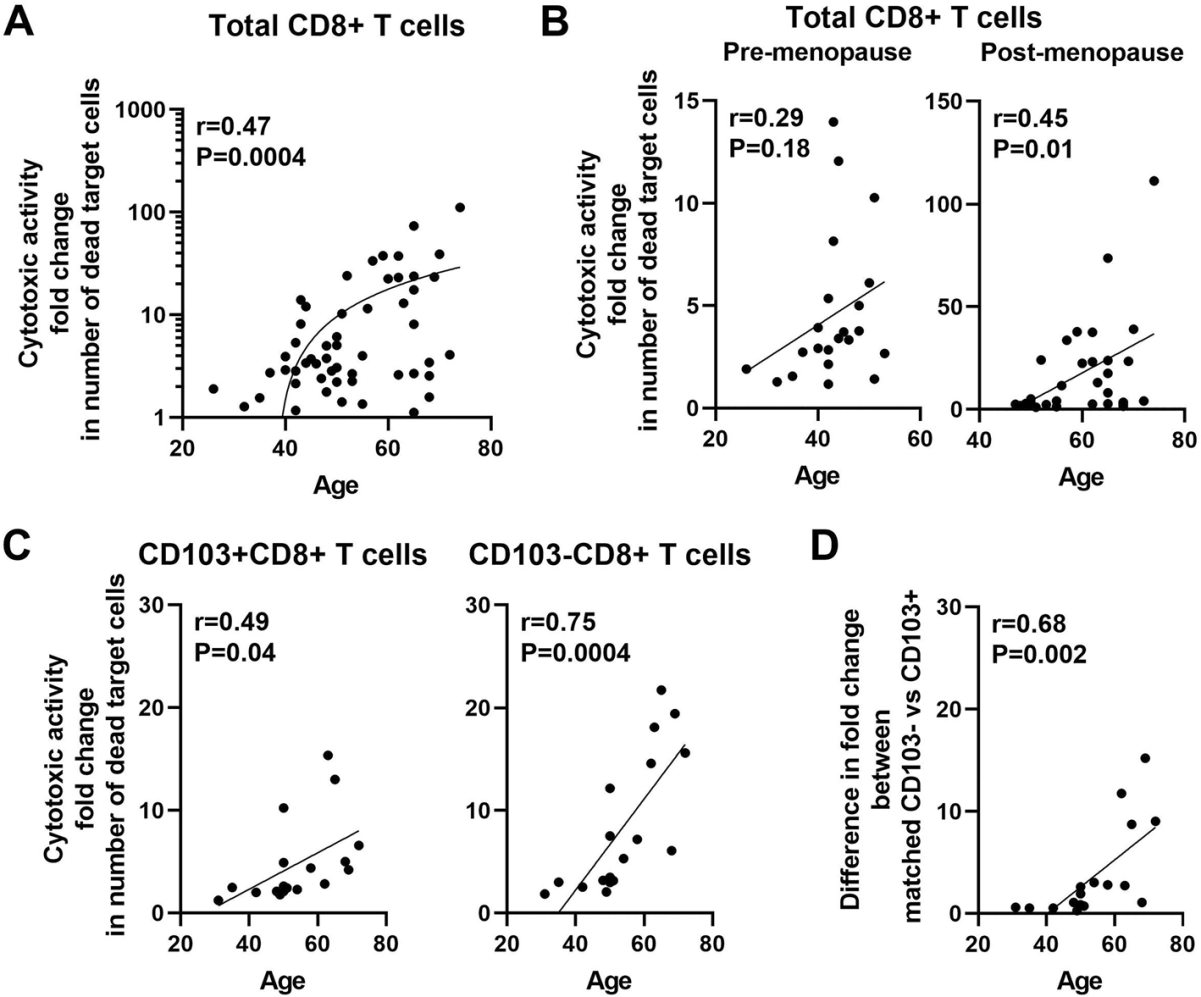
CD8+ T cells cytotoxic activity increases with age in the EM. Purified EM CD8+ T cells (or CD103+ or CD103− as indicated) were co-cultured with CFSE-stained allogeneic blood CD4+ T cells as target cells (ratio 1:1). Cytotoxicity was calculated by measuring the average number of dead target cells over the first 4 h. Graph represents the fold change in number of dead target cells in CD8+ T cells + target cells cultures compared to target cells alone. **A** Correlation between CD8+ T cells cytotoxicity and age from the entire study population (*n* = 53). **B** Correlation between CD8+ T cells cytotoxicity and age after separating pre- (left; *n* = 22) and post-menopausal cells (right; *n* = 31) women. **C** Correlation between CD103 + CD8+ T cells (left) or CD103-CD8+ T cells (right) cytotoxicity and age from the entire study population (*n* = 18). **D** Correlation between the difference of matched CD103+ and CD103-CD8+ T cells cytotoxicity and age (n = 18). Each dot represents a single patient. Spearman test

We have demonstrated previously that EM CD8+ T cell cytotoxic activity varies with stage of the menstrual cycle and increases following menopause [12, 14]. To determine the impact of age on cytotoxicity, cytotoxic killing by EM CD8+ T cells from pre- and post-menopausal women was analyzed and correlated with age. To measure cytotoxic activity, CD8+ T cells were incubated with CFSE-labelled allogeneic target cells at an Effector:Target ratio of 1:1, and the number of dead cells quantified by time-lapse imaging as described before [14, 17, 18]. Cytotoxicity was calculated by measuring the average number of dead target cells over the first 4 h and data was normalized as the fold change in number of dead target cells to be able to compare between experiments with different background mortality in the target cell alone control as previously described [14, 17, 18]. Consistent with our previous findings, the number of dead target cells in the presence of EM CD8+ T cells was significantly increased relative to target cells alone over a period of 4 h (Supplementary Fig. 2A), and EM CD8+ T cell cytotoxic killing was significantly higher in post-menopausal compared to pre-menopausal women (Supplementary Fig. 2B). When we stratified CD8+ T cell cytotoxicity as a function of age across the entire study population, we found that EM CD8+ T cell cytotoxicity increased significantly with increasing age (Fig. 2A). However, this increase in cytotoxic killing was due to the enhanced activity in CD8+ T cells from post-menopausal women with increasing age (*P* = 0.01) (Fig. 2B), while there was no effect in the pre-menopausal group (*P* = 0.18).

We then compared cytotoxic killing between EM CD103+ and CD103-CD8+ T cells and evaluated correlation with age across our entire patient population. As seen in Fig. 2C, cytotoxic killing in both the CD103+ and CD103-CD8+ T cell population significantly increased with increasing age. We have previously reported that cytotoxic killing by CD103-CD8+ T cells is significantly higher than that of CD103 + CD8+ T cells [14]. Therefore, here we investigated whether this difference was maintained with aging, and found that the difference of cytotoxic killing between CD103- and CD103+ CD8+ T cells was significantly increased with increasing age (Fig. 2D). These findings indicate that EM CD103- CD8+ T cell activity was preferentially and progressively enhanced relative to that seen with CD103 + CD8+ T cells in postmenopausal women as they age. Overall, these studies indicated that under conditions in which CD103+ and CD103-CD8+ T cell numbers decline with aging following menopause, there is a compensatory increase in CD103-CD8+ T cell cytotoxic activity.

### Aging and menopause differentially regulate intracellular cytotoxic molecules in endometrial CD8+ T cells

**Fig. 3:**
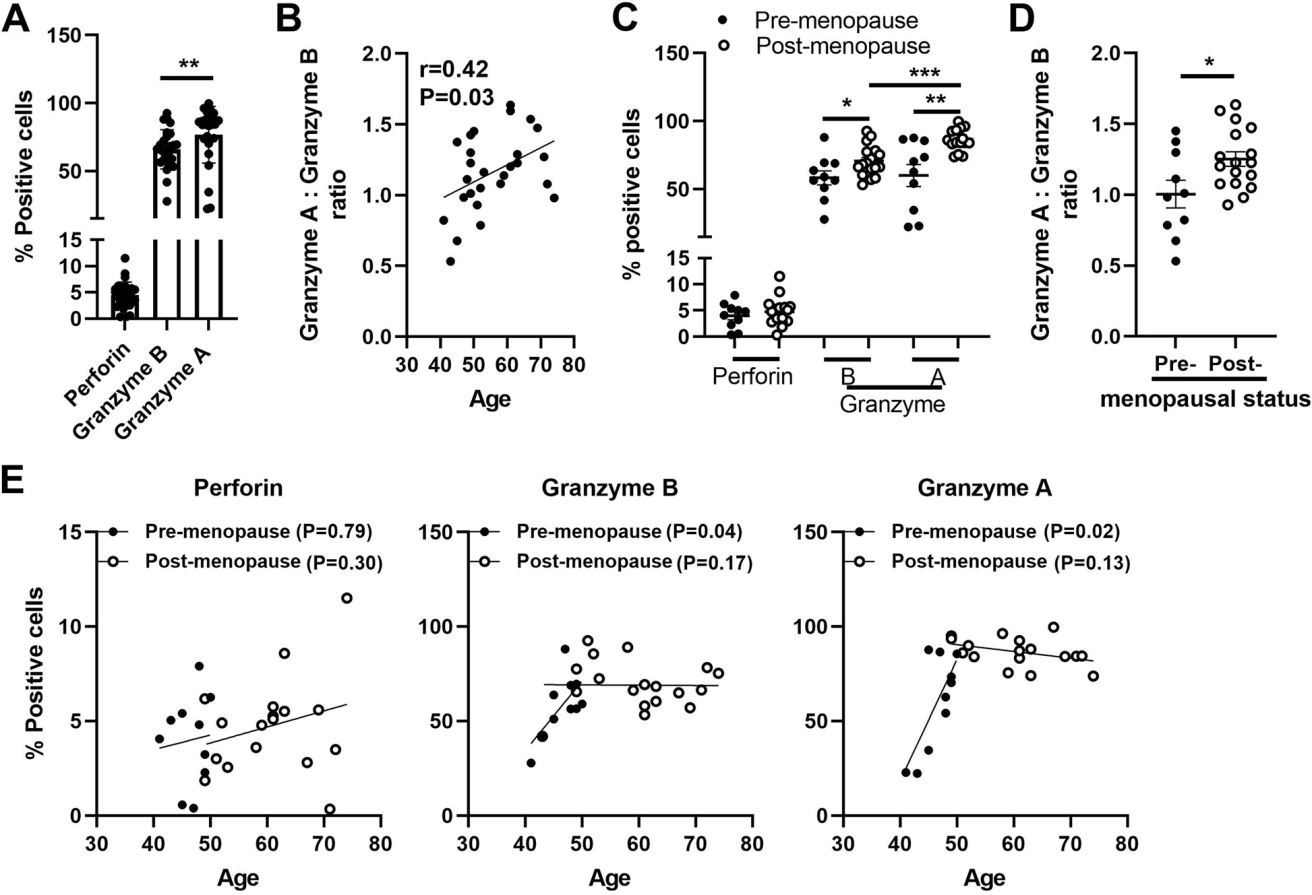
Aging and menopause differentially regulate intracellular cytotoxic molecules in EM CD8+ T cells. Mixed cell suspensions from EM tissues were stained for the intracellular cytotoxic molecules perforin, granzyme A and granzyme B for analysis by flow cytometry. **A** Percentage positive cells of perforin, granzyme A and granzyme B in EM CD8+ T cells (*n* = 27). **B** Correlation between age and the ratio of Granzyme A/Granzyme B in EM CD8+ T cells (n = 27). **C** Comparison of pre- vs. post-menopausal women percentage positive cells of perforin, granzyme A and granzyme B in EM CD8+ T cells. **D** Comparison of pre- vs. post-menopausal women the ratio of Granzyme A/Granzyme B from EM CD8+ T cells. **E** Correlation between age and the percentage positive cells of perforin (left), granzyme A (right) and granzyme B (middle) in EM CD8+ T cells from pre- and post-menopausal women. Pre-menopausal women (black circle; *n* = 10), post-menopausal women (white circle; *n* = 17). Each dot represents a different patient. Mean ± SEM are shown. *P < 0.05, **P < 0.01, ***P < 0.001; Friedman test followed by Dunns post-test (**A**), Spearman test (**B, E**). Kruskal-Wallis test followed by Dunns post-test or Friedman test followed by Dunns post-test (**C**), Mann–Whitney U-test (**D**)

To understand the mechanisms involved in the observed age-induced changes in CD8+ T cell cytotoxicity, CD8+ T cells in mixed cell suspensions from EM tissues were analyzed by flow cytometry for intracellular expression of the cytotoxic molecules perforin (PRF), granzyme A (GZMA) and granzyme B (GZMB) under resting conditions as described before [14, 17, 18]. As seen in Fig. 3A, overall, 80 and 70% of EM CD8 + T cells were GZMA+ and GZMB+ respectively. The percentage of GZMA+ and GZMB+CD8+ T cells were significantly greater than PRF + CD8+ T cells, which accounted for less than 10% of the total EM CD8+ T cell population. The percentage of GZMA+CD8+ T cells was significantly higher than the percentage of GZMB. Considering these observations, we then calculated the ratio between GZMA and GZMB expressing CD8+ T cells and detected a significant increase in GZMA/GZMB ratio with increasing age (Fig. 3B). Analysis of menopausal status demonstrated a significant increase in the percentage of GZMA+ and GZMB+CD8+ T cells (Fig. 3C) and GZMA/GZMB ratio (Fig. 3D) when post-menopausal women were compared to pre-menopausal women, with no changes detected in the percentage of PRF+ CD8+ T cells. Interestingly, when stratified by age (Fig. 3E), the percentage of GZMA+ and GZMB+CD8+ T cells increased significantly with increasing age in pre-menopausal women prior to menopause. In contrast, the percentage of GZMA+ and GZMB+CD8+ T cells in post-menopausal women remained elevated and constant with increasing age. In contrast no changes were seen in the percentage of PRF + CD8+ T cells when pre- or post-menopausal populations were analyzed with increasing age. Changes in the percentage of GZMA+ and GZMB+ cells following menopause suggest an explanation for increased cytotoxic activity of the total CD8+ T cells as women age.

### Aging differentially regulates intracellular cytotoxic molecules in endometrial CD103+ and CD103-CD8+ T cells

**Fig. 4:**
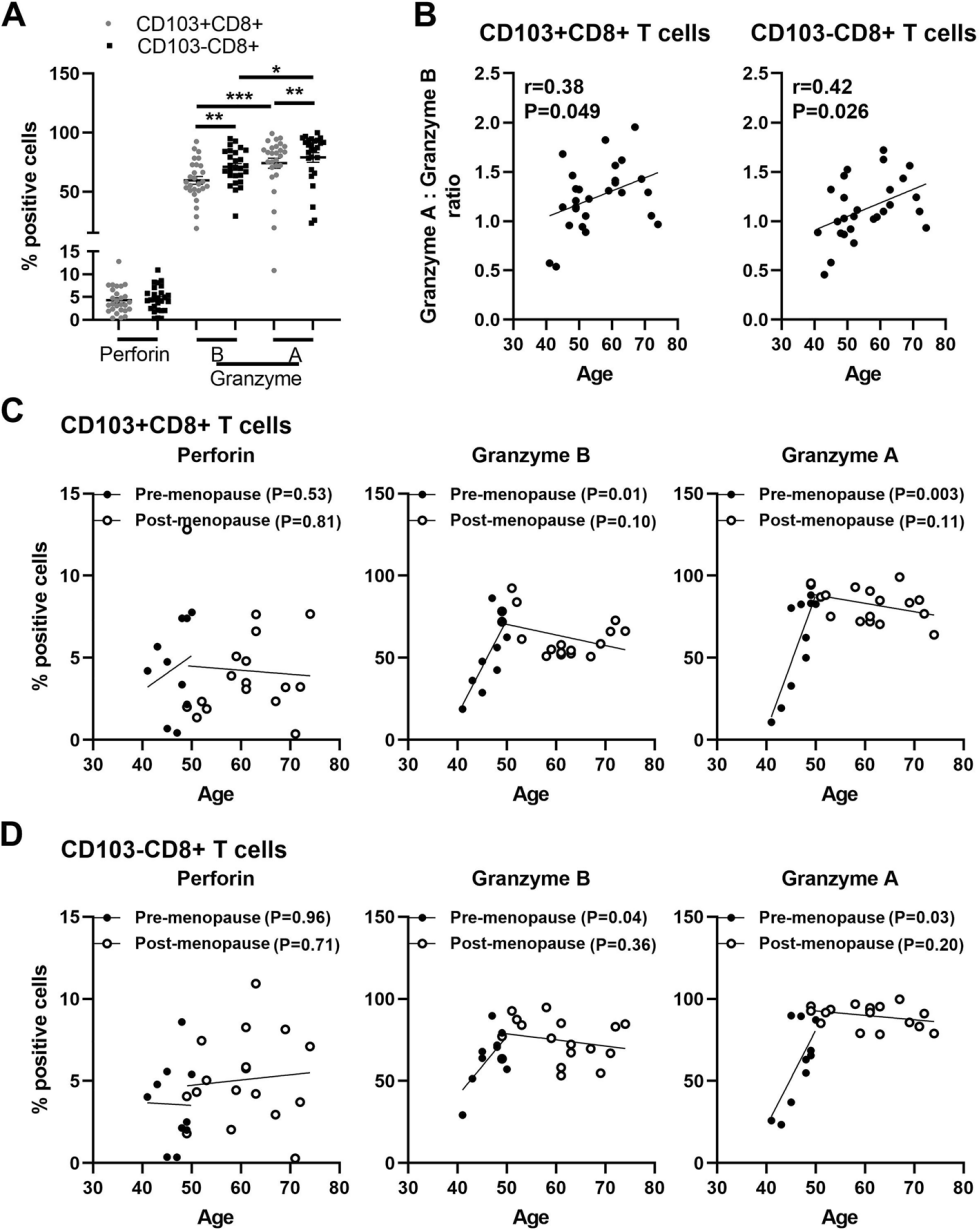
Aging differentially regulates intracellular cytotoxic molecules in EM CD103+ and CD103-CD8+ T cells. Mixed cell suspensions from EM tissues were stained for the intracellular cytotoxic molecules perforin, granzyme A and granzyme B prior to analysis by flow cytometry. **A** Percentage positive cells expressing perforin, granzyme A and granzyme B in EM CD103+ (grey circle) and CD103- (black square) CD8+ T cells (n = 27). **B** Correlation between age and the ratio of Granzyme A/Granzyme B in EM CD103+ (left) or CD103- (right) CD8+ T cells (*n* = 27). **C**, **D** Correlation between age and the percentage positive cells of perforin (left), granzyme A (right) and granzyme B (middle) in EM CD103 + CD8+ T cells (**C**) or in CD103-CD8+ T cells (**D**) from pre- (black circle; *n* = 10) and post-menopausal (white circle; *n* = 17) women. Each dot represents a different patient. Mean ± SEM are shown. *P < 0.05, **P < 0.01, ***P < 0.001; Friedman test followed by Dunns post-test (**A**), Spearman test (**B-D**)

Since we observed differential aging effects on the cytotoxic activity of CD103+ and CD103-CD8+ T cells, we next analyzed cytotoxic molecules in these populations by flow cytometry as previously described [14, 17, 18]. As seen in Fig. 4A, GZMA+ and GZMB+ were significantly more abundant in CD103- than CD103+ cells. GZMA expression was significantly greater than GZMB in both CD103+ and CD103-CD8+ T cells. The ratio of GZMA/GZMB in both CD103+ and CD103-CD8+ T cells significant increased with increasing age (Fig. 4B). When analyzed according to menopausal status (Supplementary Fig. 3A), we found that the percentage of GZMA+ cells in both the CD103+ and CD103-CD8+ T cell population increased significantly in post-menopausal women compared to pre-menopausal women. The ratio of GZMA/GZMB in CD103-CD8+ T cells, but not in CD103 + CD8+ T cells, was significantly higher in post-menopausal women compared to pre-menopausal women (Supplementary Fig. 3B).

Next, we analyzed the percent of cells positive for cytotoxic molecules as a function of age in CD103+ and CD103-CD8+ T cells. As seen in Fig. 4C and D, the percentage of GZMA+ and GZMB+ cells in both the CD103+ and CD103-CD8+ T cell population increased significantly with age in pre-menopausal women and plateaued at a high level after menopause irrespective of increasing age. In contrast, there was no effect of age on the percentage of PRF+ in either the CD103+ and CD103-CD8+ T cells from pre- or post-menopausal women. Overall, these studies demonstrate that the percentage of GZMA+ and GZMB+ EM CD8+ T cells progressively increases with increasing pre-menopausal age, reaching their peak prior to menopause, and maintaining these levels in the post-menopausal period.

### Aging and menopause differentially regulate the production of TNFα, IL-6 and IFNγ by endometrial CD8+ T cells

**Fig. 5:**
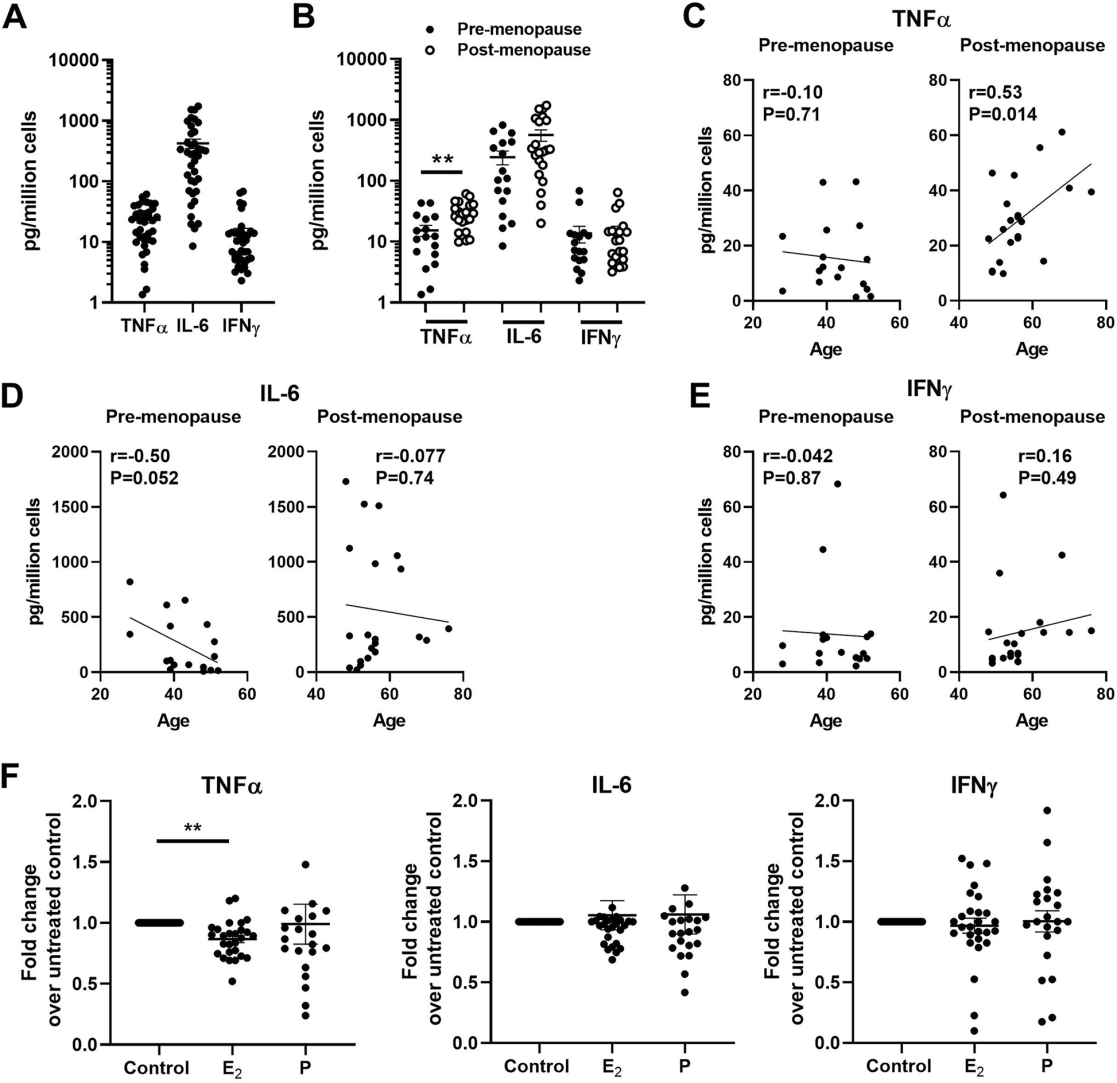
Aging and menopause differentially regulate the production of TNFα, IL-6 and IFNγ by EM CD8+ T cells. Resting purified EM CD8+ T cells were cultured with or without E_2_ (5 × 10^− 8^ M) or P (1 × 10^− 7^ M) for 48 h. The culture supernatants were collected and assayed for TNFα, IL-6 and IFNγ by Luminex assay. **A** Constitutive production of TNFα, IL-6 and IFNγ by EM CD8+ T cells after 48 h in the culture (*n* = 38). **B** Comparison of pre- (black circle; n = 17) vs. post-menopausal (white circle; *n* = 21) women constitutive production of TNFα, IL-6 and IFNγ by EM CD8+ T cells after 48 h in the culture. **C**-**E** Correlation between age and the production of TNFα (**C**), IL-6 (**D**) and IFNγ (**E**) by EM CD8+ T cells from pre- (n = 17) and post-menopausal (*n* = 21) women. **F** Effect of E_2_ or P on the production of TNFα, IL-6 and IFNγ by EM CD8+ T cells after 48 h in the culture. Graph represents the fold change in TNFα, IL-6 and IFNγ in culture supernatant following E_2_ or P treatment compared with untreated controls (E_2_: *n* = 27; P: *n* = 21). Each dot represents a single patient. Mean ± SEM are shown. **P < 0.01; Mann–Whitney U-test (**B**), Spearman test (**C**-**E**), Kruskal-Wallis test followed by Dunns post-test or Friedman test followed by Dunns post-test (**F**)

In addition to direct killing, CD8+ T cells are known to exert their actions through the secretion of cytokines and chemokines [19]. To test the hypothesis that CD8+ T cells secretion varies with age, studies were undertaken to measure TNFα, IL-6 and IFNγ production by resting CD8+ T cells as a function of aging. Purified EM CD8+ T cells were incubated for 48 h after which supernatants were collected and assayed for TNFα, IL-6 and IFNγ by Luminex assay. Data was calculated according to the cell number to compare baseline production or normalized as the fold change in baseline production to determine the effect of sex hormones. As seen in Fig. 5A, CD8+ T cells constitutively produced TNFα (23.1 ± 2.5 pg/million cells), IL-6 (421.3 ± 75.4 pg/million cells), and IFNγ (14.2 ± 2.6 pg/million cells) under resting conditions. When secretion of all three cytokines was analyzed based on menopausal status (Fig. 5B), we found significantly higher secretion of TNFα by post-menopausal CD8+ T cells compared to pre-menopausal CD8+ T cells. In contrast, there were no differences in IL-6 and IFNγ CD8+ T cell secretion between pre- and post-menopausal women. When CD8+ T cells were stratified by age, the production of TNFα (Fig. 5C) but not IL-6 (Fig. 5D) and IFNγ (Fig. 5E) increased significantly with increasing age in the post-menopausal population. There was no effect of age on the secretion of the three cytokines by pre-menopausal women.

In prior studies [17], we discovered that estradiol (E_2_) and progesterone (P) act both directly and indirectly to suppress CD8+ T cell cytotoxicity. To determine if sex hormones regulate the secretion of cytokines and chemokines, purified EM CD8+ T cells were incubated with E_2_ (5 × 10^− 8^ M) or P (1 × 10^− 7^ M) for 48 h prior to Luminex analysis. As shown in Fig. 5F, E_2_ but not P significantly suppressed TNFα secretion by EM CD8+ T cells, with no effect of either sex hormone on IL-6 or IFNγ secretion. Overall, these studies indicate that following menopause and with increasing post-menopausal age, constitutive secretion of TNFα by CD8+ T cells increases in response to the absence of E_2_, possibly to enhance immune protection in the EM, at a time when protection is declining.

### Aging and menopause differentially regulate the production of TNFα, IL-6 and IFNγ by endometrial CD103+ and CD103-CD8+ T cells after activation

**Fig. 6:**
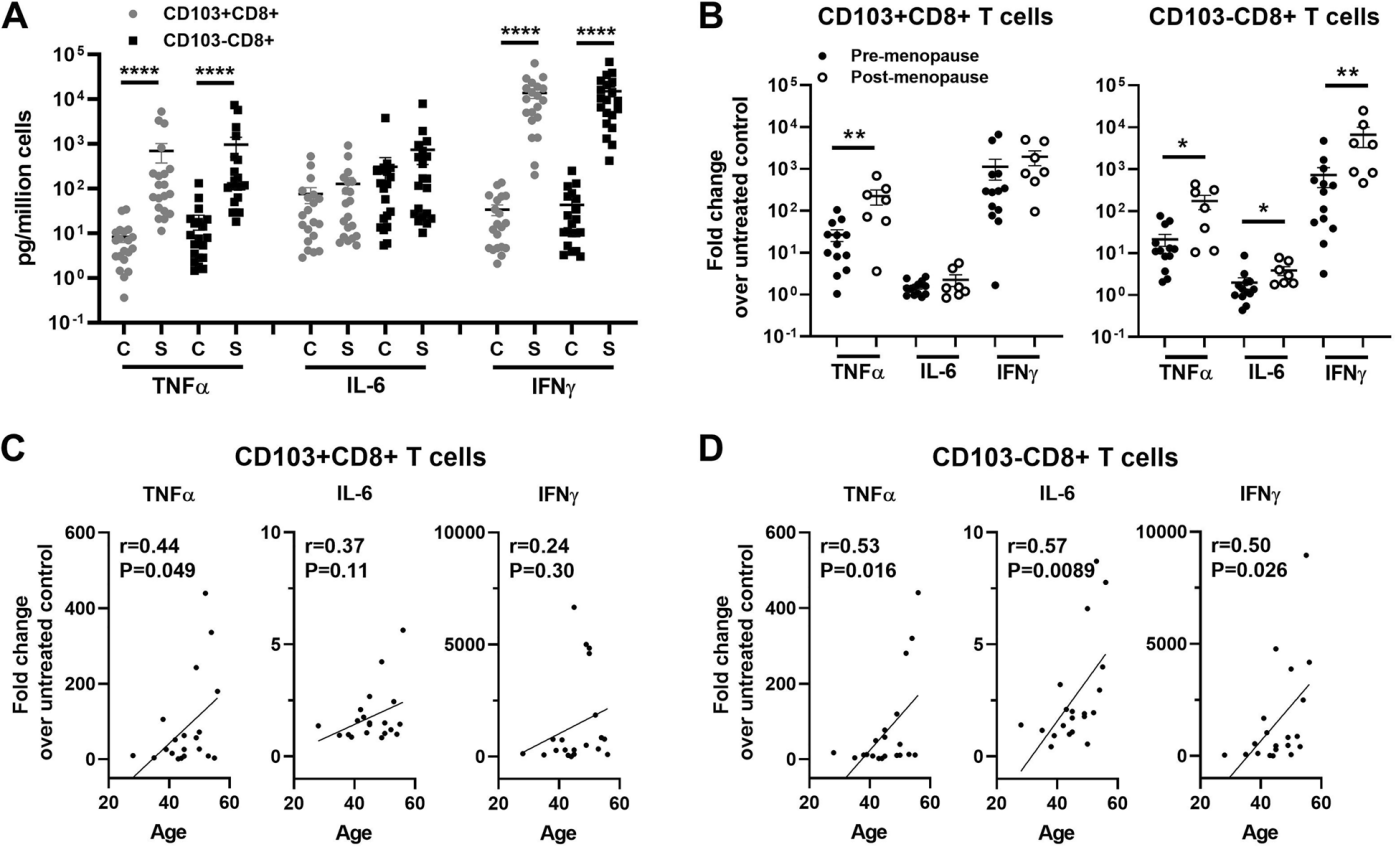
Aging and menopause differentially regulate the production of TNFα, IL-6 and IFNγ by EM CD103+ and CD103-CD8+ T cells after activation. Purified EM CD103+ and CD103-CD8+ T cells were stimulated with PMA (100 ng/ml) and ionomycin (2 μM) for 24 h. The culture supernatants were collected and assayed for TNFα, IL-6 and IFNγ by Luminex assay. **A** Production of TNFα, IL-6 and IFNγ by EM CD103+ (grey circle) and CD103- (black square) CD8+ T cells after 24 h stimulation (*n* = 20). C represents untreated control. S represents stimulation with PMA/ionomycin. **B** Comparison of pre- (black circle; *n* = 13) vs. post-menopausal (white circle; *n* = 7) women production of TNFα, IL-6 and IFNγ by EM CD103+ (left) and CD103- (right) CD8+ T cells after 24 h stimulation. **C**, **D** Correlation between age and the production of TNFα, IL-6 and IFNγ by EM CD103 + CD8+ T cells (**C**) and CD103-CD8+ T cells (**D**) after 24 h stimulation. Graphs of (**B**-**D**) represent the fold change in TNFα, IL-6 and IFNγ in culture supernatant following stimulation compared with untreated controls (*n* = 20). Each dot represents a single patient. Mean ± SEM are shown. *P < 0.05, **P < 0.01, ****P < 0.0001; Friedman test followed by Dunns post-test (**A**), Kruskal-Wallis test followed by Dunns post-test (**B**), Spearman test (**C**, **D**)

In previous studies, we identified differences after activation of resident and non-resident CD8+ T cells and found that EM CD103 + CD8+ T cells had significantly higher degranulation following activation compared to CD103-CD8+ T cells, but that higher CD103+ CD8+ T cell degranulation did not result in the increased release of cytotoxic molecules [14]. Whether aging affects the secretion of TNFα, IL-6, or IFNγ by CD103+ and CD103-CD8+ T cells remains unknown. We therefore analyzed the secretions of all three cytokines following activation and stratified these results according to menopausal status and age. Purified EM CD103+ and CD103-CD8+ T cells were activated with PMA (100 ng/ml) and ionomycin (2 μM) for 24 h as previously described [14]. The culture supernatants were collected and assayed for TNFα, IL-6 and IFNγ by Luminex assay. Data were calculated according to the cell number and normalized as the fold change in baseline production to determine response to PMA stimulation. As shown in Fig. 6A, secretion of TNFα and IFNγ increased significantly in both CD103+ and CD103-CD8+ T cells after 24 h stimulation with PMA, with no change in secretion of IL-6. When stratified by menopausal status (Fig. 6B), secretion of TNFα by both CD103+ and CD103-CD8+ T cells significantly increased in post-menopausal women. In contrast, IL-6 and IFNγ secretion by CD103-CD8+ T cells increased in post-menopausal but not in CD103 + CD8+ T cells.

We then stratified secretion of TNFα, IL-6, and IFNγ by both activated CD103+ and CD103-CD8+ T cells with increasing age. As seen in Fig. 6C and D, TNFα, IL-6 and IFNγ secretion by CD103-CD8+ T cells significantly increased with increasing age. In contrast, only TNFα secretion increased with increasing age in CD103 + CD8+ T cells. Overall, these studies indicate that with age, CD8+ T cells are able to respond to potential challenges with increased secretion of pro-inflammatory mediators that are capable of mounting immune protection.

## Discussion

The present study demonstrates that immunological changes continue to occur in the EM CD8+ T cell population with increasing age following menopause. Both the total number of CD8+ T cells, as well as the number of CD103+ and CD103-CD8+ T cells, declined significantly with increasing age in the years following menopause. Despite this decline in cell numbers, cytotoxic killing by both CD103+ and CD103-CD8+ T cells significantly increased with increasing age. The percentage of GZMA+ and GZMB+ cells in CD8+ T cell population significantly increased with menopause and remained elevated as women aged. Constitutive secretion of TNFα by EM CD8+ T cells significantly increased with increasing age after menopause and could be suppressed by treatment with E_2_. There were no effects of age and menopausal status on constitutive secretion of IFNγ and IL-6. However, following stimulation with PMA, the secretion of TNFα, IL-6, and IFNγ all significantly increased with increasing age in CD103- but not CD103 + CD8+ T cells. These findings suggest that in the EM, CD103+ and CD103-CD8+ T cell compensation occurs to sustain mucosal immune protection. Overall, our results highlight the protective effect of CD8+ T cells in the EM environment and the impact of aging following menopause on immune protection.

To more fully understand the dynamics of cell-mediated immunity in the years following menopause, we examined the number of CD8+ T cells in the EM as a function of age and showed, for the first time, that total CD8+ T cell numbers, as well as CD103+ and CD103-CD8+ T cell numbers, decreased with increasing age in both our total population (26–81 years) as well as in the post-menopausal population. Whether this decline is due to altered recruitment into the EM mucosa is unknown. We and others have found that in pre-menopausal women the total leucocyte population in the EM remains either unchanged or increases slightly during the menstrual cycle, and then declines following menopause [11]. We have also shown that EM dendritic cell (DC) numbers decline with increasing age in a combined population of pre- and post-menopausal women [13]. However, studies with blood CD8+ T cells have shown either a decrease in naïve CD8+ T cells with aging [20] or an increase in effector memory CD8+ T cell subsets with no changes in the total number of T cells [21]. Since EM CD8+ T cells are over 95% memory T cells [1, 3], this may suggest that the decrease in CD103-CD8+ T cells in the EM observed by us could be due to impaired recruitment from blood, potentially via altered chemokine production by other EM cells, rather than a decrease in CD8+ T cell numbers in circulation. For example, we have shown that EM epithelial cells from post-menopausal women decrease their production of the chemokine/antimicrobial Secretory Leucocyte Protease Inhibitor (SLPI) by approximately 80% compared to pre-menopausal women [22]. Whether changes in baseline secretion of T cell chemokines such as CCL2, CCL21, RANTES, and SDF-1α by EM cells occur with increasing age remains to be determined but could partly explain why CD103-CD8+ T cells decline in the aging EM.

Beyond CD8+ T cell recruitment, other mechanisms such as retention and local proliferation are likely to influence the decline in cell numbers in the EM [23]. Others have demonstrated the expression of adhesion proteins on epithelial cells and endothelial cells [24, 25]. These bind to the migrating lymphocytes, directing their entry and retention into tissues [26]. For example, E-cadherin is essential for the retention of CD103+ cells in mucosal tissues [23]. We have shown that EM DCs from post-menopausal women were significantly more effective at inducing CD103 expression on allogeneic naive CD8+ T cells than DCs from pre-menopausal women and that the proportion of EM CD103+ T cells, as a fraction of the total number of CD8+ T cells, is higher in post-menopausal women compared to pre-menopausal women [13]. Consistent with this observation is our finding that the proportion of CD103 + CD8+ T cells as a percentage of the total number of CD8+ T cells increases from 41 to 49% between pre- and post-menopausal women respectively. This suggests that the EM CD103 + CD8+ T cells may be compensating for their declining overall numbers by increasing their interactions with epithelial cells. However, whether epithelial expression of E-cadherin, the ligand for CD103, changes with age is unknown. If E-cadherin expression is declining on epithelial cells with increasing age, then the increase in CD103 expression may not be sufficient to compensate and thus result in an overall decrease in EM CD103 + CD8+ T cells. Further studies are needed to understand the complexities in the human EM that result in a progressive decline in CD8+ T cell numbers as women age following menopause.

As discussed above, CD103 is an important marker for tissue residency; at present, it is unclear as to what drives the decrease in CD103 expression on EM CD8+ T cells. Multiple factors such as TGFβ can influence the expression of CD103 on CD8+ T cells. EM TGFβ expression varies across the menstrual cycle and with menopause, modulating multiple aspects of EM immune function [27]. We have shown that plasma membrane bound TGFβ on the surface of EM DCs induces the expression of CD103 on blood CD8+ T cells [13]. However, TGFβ1 expression increases in the post-menopausal EM versus the pre-menopausal EM [28], suggesting that decreased CD103 expression is not driven by TGFβ levels in the aging EM. Further studies are required to understand the regulatory control of CD103 expression on CD8+ T cells in the post-menopausal EM.

Sex hormone levels during the menstrual cycle influence both cell chemotaxis, retention, and the distribution of immune cells in the FRT [1, 29]. Whether sex hormones can directly induce the chemotaxis of CD8+ T cells in the non-pregnant human EM is unclear, but studies in animal models have shown no effect of estradiol [30]. However, sex hormones likely act via intermediate cells to alter the balance of the chemotactic signals in the EM. For example, when incubated with estradiol for 24-48 h, we found that uterine epithelial cells increased secretion of SLPI (four-fold) and mRNA expression of HBD2 (five-fold), both of which are known to be chemotactic [31]. Whether changes in chemokine production due to the loss of sex hormones following menopause accounts for the continued decline in CD8+ T cell numbers remains to be determined. Alternatively, sex hormones may regulate the expression of adhesion molecules such as E-cadherin and alter the retention of cells in the EM mucosa. For example, estradiol is known to upregulate the expression of E-cadherin on prostate cancer cells [32]. Therefore, it is plausible that the presence of estradiol in pre-menopausal women may lead to the upregulation of E-cadherin expression on EM epithelial cells as a potential way of maintaining CD8+ T cells in the mucosa. Finally, the distribution of CD8+ T cells within the EM mucosa may be under hormonal control as well. We previously demonstrated that EM lymphoid aggregates, consisting primarily of CD8+ T cells are largest during the secretory phase of the menstrual cycle, before being dispersed during menses and following menopause [15]. Since the secretory phase is characterized by high levels of both progesterone and estradiol, this strongly suggests that the distribution CD8+ T cells in the EM is affected by hormone levels. Precisely how sex hormones affect cell retention and distribution is unknown but is likely to involve both adhesion markers and chemokines that direct CD8+ T cells to specific locations in the EM.

Our studies demonstrate that direct cytotoxic activity per cell increases with age despite an overall decline in CD8+ T cell numbers. This is the first demonstration that CD8+ T cell cytotoxic killing in the post-menopausal FRT changes with increasing age. We have previously demonstrated that CD8+ T cell cytotoxicity in the EM is suppressed during the menstrual cycle and increased following menopause [12, 14]. In addition, we have shown that EM tumors suppress this enhanced cytotoxicity in post-menopausal women [18]. However, regulation of CD8+ T cytotoxic activity in the years following menopause remains an unanswered question. Mechanistically, we have previously shown that estradiol and progesterone act both directly and indirectly to suppress cytotoxic activity of post-menopausal CD8+ T cells [17]. Whereas estradiol directly suppressed CD8+ T cell cytotoxicity, progesterone acted indirectly by upregulating EM epithelial cells secretion of TGFβ which suppressed cytotoxic activity as well as perforin and granzyme expression in CD8+ T cells [14, 17]. Since the majority of our previous hormone treatment studies were carried out using EM CD8+ T cells from post-menopausal women, we hypothesize that the enhanced cytotoxic activity observed after menopause is due to the decline in sex hormones that accompany the post-menopausal years. What remains to be determined is why, rather than plateauing, cytotoxic activity continues to rise as women age in the decades following menopause. One explanation for this increase in cytotoxicity could be due to the loss of self-tolerance and defective surveillance by CD8+ T cells with increasing age [33, 34]. Previous studies have shown that inflammaging, which is characteristic of older individuals, drives CD8+ T cells towards senescence [35, 36]. Senescent CD8+ T cells have enhanced cytotoxicity that could lead to increased pathology. Interestingly, Cytomegalovirus (CMV) infection (whose prevalence increases with age) has been associated with immunosenescence and with accumulation of T cells with high cytotoxic potential in peripheral blood, particularly in men [37]. The extent to which CMV infection may affect senescence of CD8+ T cells in the EM is completely unknown and would be an important aspect to address in the future. Furthermore, future studies will need to determine whether enhanced EM CD8+ T cell cytotoxicity in post-menopausal women is beneficial or detrimental to health.

The percentage of total CD8+ T cells that were GZMA+ and GZMB+ increased significantly at the onset of menopause and remained elevated as women aged, suggesting that the presence of sex hormones in pre-menopausal women suppress the numbers of GZMA+ and GZMB+CD8+ T cells, and that the reduction of sex hormone production following menopause directs EM CD8+ T cells towards a GZMA+ and GZMB+ phenotype. In earlier studies, we found that the degranulation capacity of CD103- and CD103 + CD8+ T cells increased following menopause [14] suggesting that these cells may be more responsive to activation signals. Taken together, these findings suggest that compared to pre-menopausal cells, both CD103+ and CD103-CD8+ T cells following menopause are predisposed to mount a cytotoxic response, despite undergoing a decline in overall numbers. Others have implicated additional members of the GZM family, in particular GZMK, in the enhanced cytotoxic response, that leads to increased senescence in adjacent cells [38]. Since the percent of PRF + CD8+ T cells remains low both prior to and following menopause, questions remain regarding the mechanisms involved in increased cytotoxic activity and the protective effects of CD8+ T cells as women age, since both PRF and GZM are essential components of the primary apoptosis pathway necessary for eliminating cells [39]. Further studies are needed to more fully understand mechanisms involved in PRF and GZM changes that occur with aging following menopause.

An unexpected finding in our studies was that TNFα, IL-6 and IFNγ secretion by CD103-CD8+ T cells significantly increased in post-menopausal women and with increasing age in response to PMA activation. To the best of our knowledge, our findings are the first to demonstrate a progressive increase in induced cytokine production in the EM by CD8+ T cells with increasing age. Since TNFα, IL-6 and IFNγ are inflammatory cytokines important for resistance to infection and cancers, our findings suggest that under acute conditions, in addition to enhanced cytotoxic killing that protects against potential pathogens, post-menopausal CD8+ T cells have increased signaling capacity to upregulate adaptive immune protection in the EM. One caveat with our activation protocol is that we utilized PMA with ionomycin to stimulate EM CD8+ T cells. This protocol bypasses the T-cell receptor (TCR), which is the primary T cell activation pathway in vivo. PMA with ionomycin elicits a more pronounced inflammatory response with a large number of responding cells compared to anti-CD3/CD28 stimulation of CD8+ T cells via the TCR [40]. Since PMA with ionomycin stimulation of CD8+ T cells tends to induce a more potent inflammatory response compared to anti-CD3/CD28, it will be important for future studied to stimulate the CD8+ T cells via the TCR, to determine how aging affects the inflammatory response via this pathway. Nevertheless, our findings provide valuable information about how CD8+ T cell responses may be intrinsically affected by aging. Since PMA directly activates the protein kinase C (PKC) signaling pathway [41], our data would suggest that this pathway is more strongly activated with aging through yet unknown mechanisms. Previous studies have shown changes in the epigenetic signature of peripheral CD8+ T cells with aging [42–44], including epigenetic modifications of cytotoxic genes [20]. Whether EM CD8+ T cells also undergo epigenetic changes following menopause is unknown, but it is likely given the age-associated epigenetic modification occurring in blood CD8+ T cells. Future studies are needed to define epigenetic changes in EM CD8+ T cells and how they may control cytotoxic responses.

What remains unclear is the effect of TNFα, IL-6 and IFNγ in adjacent EM tissue as well as the mechanisms through which CD8+ T cells maintain a balance between their beneficial effects and detrimental pro-inflammatory activities. Increased production of inflammatory cytokines is characteristic of inflammaging [45, 46], which increases senescence in adjacent cells (senescence-associated secretory phenotype), and further promotes tissue inflammation. In the peripheral circulation, inflammaging has been associated with increased levels of certain cytokines including TNFα and IL-6. Senescent T cells are characterized by increased secretion of a spectrum of pro- and anti-inflammatory cytokines [38]. Furthermore, inflammatory cytokines, particularly TNFα, have been shown to weaken barrier function of EM epithelial cells [47], potentially weakening innate immune protection against external pathogens. Since it is unclear as to the extent to which EM CD8+ T cells demonstrate a senescent phenotype, further studies are needed to determine whether CD8+ T cell secretion of TNFα, IL-6 and IFNγ in the FRT is self-limiting to ensure beneficial responses without being detrimental to health.

## Conclusions

Our findings demonstrate a previously unrecognized shift in CD8+ T cell function with aging in the uterine EM in the years following menopause. Despite a decline in resident and non-resident CD8+ T cells, both possess enhanced cytotoxic capacity, suggesting that a decline in CD8+ T cell numbers with age does not necessarily correlate with decreased protection against pathogens. Taken together with our past studies, our results further demonstrate that the post-menopausal EM is not a static environment, and that its immunological function and capacity continually undergoes changes with increasing age. Understanding the underlying factors and mechanisms involved in regulating cell-mediated protection of the EM by CD8+ T cells will contribute to the foundation of information essential for developing therapeutic tools to protect women against gynecological cancers and sexually transmitted infections as they age in the years following menopause.

## Methods

### Study subjects

Endometrial (EM) tissues were obtained following hysterectomy surgery. Studies were performed with Dartmouth College Institutional Review Board (IRB) approval as well as the Committee for the Protection of Human Subjects (CPHS), Dartmouth-Hitchcock Medical Center (DHMC), and with written informed consent obtained from the patients before surgery. All investigations were conducted according to the principles expressed in the Declaration of Helsinki. Indications for surgery were benign conditions such as fibroids and prolapse (age from 26 to 81). EM tissue samples selected were distant from the sites of pathology and without pathological lesions as determined by a pathologist from DHMC. Women were not on oral contraceptives or post-menopausal hormone therapy prior to hysterectomy. Menopausal status was determined by a pathologist based on the histological evaluation of sections of the EM (endometrial dating). Post-menopausal status was defined as an atrophic EM. Information regarding genital infections was not available.

### Tissue processing

EM tissues were transferred to the laboratory immediately after surgery and processed as previously described [13, 14, 17, 48]. Average tissue weight obtained was 4.0 ± 2.9 g. Tissues were rinsed with HBSS (Hanks balanced salt solution) supplemented with phenol red, 100 U/ml penicillin, 100 μg/ml streptomycin (all Thermo Scientific Hyclone, Logan, UT), and 0.35 mg/ml NaCO_3_ (Fisher Scientific, Pittsburgh, PA). Tissues were then minced under sterile conditions into 1–2 mm fragments and digested using an enzyme mixture containing 0.05% collagenase type IV (Sigma-Aldrich, St. Louis, MO) and 0.01% DNAse (Worthington Biochemical, Lakewood, NJ) for 1 h at 37 °C. Type IV collagenase was selected based on studies to ensure non-cleavage of surface markers [49, 50]. After digestion, cells were dispersed through a 250 μm nylon mesh filter (Small Parts, Miami Lakes, FL) followed by sequential filtration of the flow-through through 40 and 20 μm nylon mesh filters (Small Parts). Epithelial cell sheets were retained on the filters, while stromal cells passed through. Stromal cells were washed, erythrocytes lysed, and dead cells removed using the Dead cell removal kit (Miltenyi Biotec, Auburn, CA) according to manufacturer instructions. The resulting mixed cell suspension, consisting of immune cells and stromal fibroblasts, was used for flow cytometric analysis and further CD8+ T cell purification.

### Preparation of EM CD8+ T cells

Following removal of dead cells, CD8+ T cells were isolated using negative magnetic bead selection with the CD8+ T cell isolation kit (Miltenyi Biotec) following instructions with minor modifications. This negative selection protocol delivers untouched CD3 + CD8+ T cells. Additionally, anti-fibroblast microbeads (Miltenyi Biotec) were added in combination with the microbeads supplied with the kit to ensure depletion of stromal fibroblasts present in the mixed cell suspension as described before for CD4 selection [49]. After two rounds of negative selection, purity of the CD8+ T cell population was higher than 90%, with approximately 2% contamination with non-immune cells, 2% CD3- cells and 1–2% contamination with CD4+ T cells [14]. Following isolation, viable purified CD8+ T cells were counted using trypan blue (Fisher Scientific) by hemocytometer and resuspended in X-VIVO 15 without Phenol Red Media (Lonza, Walkersville, MD), prior to use in cytotoxicity assays, Luminex assays or for further CD103+ and CD103-CD8+ T cells separation.

### Preparation of EM CD103+ and CD103-CD8+ T cell

We have developed, an optimized protocol to isolate CD103+ and CD103 − CD8+ T cells from EM tissues [14]. Purified EM CD8+ T cells were incubated with CD103-PE antibody (Miltenyi Biotec) for 10 min, followed by incubation with anti-PE ultra-pure beads (Miltenyi Biotec) to separate CD103+ cells by positive magnetic separation, and CD103− by negative selection. By using sequential magnetic bead selection, we obtained two purified EM CD8+ T cell populations from the same patients (CD103+ and CD103−), with a purity range between 90 and 96% [14]. Following isolation, viable EM CD103+ and CD103-CD8+ T cells were counted using trypan blue by hemocytometer and resuspended in X-VIVO 15 Media, prior to further use.

### Preparation of blood CD4+ T cells

Blood Leuko Paks from women were obtained from our IRB-approved collection facility at DHMC. Blood donors were anonymous, no information regarding age or hormonal status was available and only female donors were used in this study. Blood CD4+ T cells were purified using negative magnetic beads selection with a CD4+ T cell isolation kit (Miltenyi Biotech) following isolation of peripheral blood mononuclear cells (PBMC) by standard Ficoll density gradient centrifugation [49, 51]. Freshly isolated blood CD4+ T cells were stained with CFSE (Cell Division Tracker Kit; BioLegend, San Diego, CA) as recommended by the manufacturer. The cells were then resuspended in X-VIVO 15 Media as allogeneic target cells prior to cytotoxicity assays.

### Cytotoxicity assay

Cytotoxic activity of purified EM CD8+ T cells was evaluated as described previously using an allogeneic cytotoxicity assay [14, 17]. Purified EM CD8+ T cells (or CD103+ or CD103− as indicated) were co-cultured with CFSE-stained allogeneic blood CD4+ T cells in X-VIVO 15 media, at an Effector:Target ratio of 1:1, in 96-well plates. Cytotox red (IncuCyte Cytotox Red, Essen Bioscience, Ann Arbor, MI) was added to the media to stain dead cells. Plates were imaged every 10 min using the IncuCyte Zoom system (Essen Bioscience), and dead target cells were automatically quantified over time as double green (CFSE) and red (Cytotox) stained cells. Cytotoxicity was calculated by measuring the average number of dead target cells over the first 4 h and normalized the data as the fold change in number of dead target cells to be able to compare between experiments with different background mortality in the target cell alone control.

### Flow cytometry

Mixed cell suspensions were stained for surface markers with combinations of the following antibodies: CD45-AF700 (HI30, Cat # 304024, BioLegend), CD3-APC/Cy7 (OKT3, Cat # 317342, BioLegend), CD103-BV711 (Ber-ACT8, Cat # 350222, BioLegend), CD4-PE/Cy5.5 (RM4–5, Cat # 350042, eBioscience, San Diego, CA), CD8-BUV395 (RPA-T8, Cat # 563795, BD Biosciences). Following surface staining, cells were fixed and permeabilized with Cytofix/cytoperm kit (BD Biosciences) according to instructions to detect perforin, granzyme A and B cells. Intracellular staining of perforin, granzyme A and B was done using combinations of the following antibodies: anti-human Perforin-PE/Dazzle 594 (dG9, Cat # 308132, BioLegend), Granzyme A-AF647 (CB9, Cat # 507214, BioLegend) and Granzyme B-BV421 (GB11, Cat # 563389, BD Biosciences). Analysis was performed on BioRad ZE5 flow cytometers (Bio-Rad, Hercules, CA) using Everest software, and data analyzed with FlowJo software (Tree Star, Inc. Ashland, OR). Expression of intracellular markers was measured by the percentage of positive cells. The gating strategy used for the expression of intracellular perforin, granzyme A and B analysis can be found in Supplementary Fig. 4.

### Hormone preparation

17β-estradiol (E_2_, Calbiochem, Gibbstown, NJ) and progesterone (P, Calbiochem) were dissolved in 100% ethanol for an initial concentration of 1 × 10^− 3^ M, evaporated to dryness and suspended in X-VIVO 15 without Phenol Red media supplemented with 10% charcoal dextran-stripped human AB serum (Valley Biomedical) to a concentration of 1 × 10^− 5^ M. Further dilutions were made to achieve final working concentration, and cells were treated with 5 × 10^− 8^ M E_2_ or 1 × 10^− 7^ M P. Both are standard hormone treatment concentrations used by our laboratory and each is within the physiological range of hormone concentrations in the FRT [52]. As a control, an equivalent amount of ethanol without dissolved hormone was initially evaporated.

### Luminex assay

Isolated purified EM CD8+ T cells or purified CD103+ and CD103-CD8+ T cells were plated (50,000–200,000 cells/well) in round bottom ultra-low attachment 96-well plates (Corning, Corning, NY) in X-VIVO 15 without Phenol Red media supplemented with 10% charcoal dextran-stripped human AB serum. Purified CD8+ T cells were treated with or without E_2_ (5 × 10^− 8^ M) or P (1 × 10^− 7^ M) for 48 h. Purified CD103+ and CD103-CD8+ T cells were activated with phorbol 12- myristate 13-acetate (PMA) (100 ng/ml, Abcam) and ionomycin (2 μM, Calbiochem) for 24 h. The culture supernatants were collected and stored at − 80 °C until analysis. TNFα, IL-6 and IFNγ were measured using Millipore human cytokine multiplex kits (EMD Millipore. Corporation, Billerica, MA) according to the instructions. Signal was measured using the Bio-Plex array reader (Bio-Rad). Bio-Plex Manager software with five-parametric-curve fitting was used for data analysis. Data were calculated according to the cell number to compare baseline production or normalized as the fold change in baseline production to determine responding to hormone treatment or PMA stimulation.

### Statistics

Data analysis was performed using the GraphPad Prism 9 (GraphPad Software, San Diego, CA). A two-sided *P* value < 0.05 was considered statistically significant. Comparison of two groups was performed with the nonparametric Mann–Whitney U-test. Comparison of three or more groups was performed applying the non-parametric Kruskal-Wallis test or the paired Friedman test followed by Dunns post-test. Correlation analyses were performed applying nonparametric Spearman test.

## Supplementary Information

**Additional file 1: Supplementary Fig. 1.** Menopause differentially regulates CD8+ T cell numbers in EM. (A) Comparison of pre- (black circle; *n* = 35) vs. post-menopausal (white circle; *n* = 33) women number of CD8+ T cells recovered per gram of EM tissue after magnetic bead isolation. (B) Comparison of pre- (black circle; *n* = 21) vs. post-menopausal (white circle; *n* = 17) women number of CD103+ (left) or CD103- (right) CD8+ T cells recovered per gram of EM tissue after magnetic bead isolation. Each dot represents a single patient. Mean ± SEM are shown. ***P < 0.001; Mann–Whitney U-test.

**Additional file 2: Supplementary Fig. 2.** Menopause differentially regulates CD8+ T cells cytotoxic activity in EM. (A) Representative example of dead target cells kinetics with purified EM CD8+ T cells + target cells (ratio 1:1; dark circle) or target cells alone (open circle) over a period of 4 h. (B) Comparison of pre- (black circle; *n* = 22) vs. post-menopausal (white circle; *n* = 31) women EM CD8+ T cells cytotoxic activity. Graph represents the fold change in number of dead target cells in CD8+ T cells + target cells cultures compared to target cell alone. Each dot represents a single patient. Target cells are allogeneic blood CD4+ T cells. Mean ± SEM are shown. *P < 0.05; Mann–Whitney U-test.

**Additional file 3: Supplementary Fig. 3.** Menopause differentially regulates CD103+ and CD103-CD8+ T cells intracellular cytotoxic molecules in EM. Mixed cell suspensions from EM tissues were stained for the intracellular cytotoxic molecules perforin, granzyme A and granzyme B for analysis by flow cytometry. (A) Comparison of pre- vs. post-menopausal women percentage positive cells of perforin, granzyme A and granzyme B in EM CD103+ (left) or CD103- (right) CD8+ T cells. (B) Comparison of pre- vs. post-menopausal women the ratio of Granzyme A/Granzyme B from EM CD103+ (left) or CD103- (right) CD8+ T cells. Pre-menopausal women (black circle; *n* = 10), post-menopausal women (white circle; *n* = 17). Each dot represents a different patient. Mean ± SEM are shown. *P < 0.05, **P < 0.01, ***P < 0.001; Kruskal-Wallis test followed by Dunns post-test or Friedman test followed by Dunns post-test (A), Mann–Whitney U-test (B).

**Additional file 4:** **Supplementary Fig. 4.** Gating strategy used for analysis the expression of intracellular perforin, granzyme A and B on CD8+ T cells, or CD103+ and CD103-CD8+ T cells in the mixed cell preparation from endometrium tissue.

## Data Availability

The data that support the findings of this study are available from the corresponding author upon reasonable request.

## Abbreviations

CCL2: Chemokine (C-C motif) ligand 2
CCL21: Chemokine (C-C motif) ligand 21
CFSE: Carboxyfluorescein succinimidyl ester
CMV: Cytomegalovirus
DC: Dendritic cell
E_2_: Estradiol
EM: Endometrium, endometrial
FRT: Female reproductive tract
GZMA: Granzyme A
GZMB: Granzyme B
GZMK: Granzyme K
IL-6: Interleukin-6
IFNγ: Interferon gamma
P: Progesterone
PBMC: Peripheral blood mononuclear cells
PKC: Protein kinase C
PMA: Phorbol 12- myristate 13-acetate
PRF: Perforin
RANTES: Chemokine (C-C motif) ligand 5, CCL5
SDF-1α: Stromal cell-derived factor-1 alpha
SLPI: Secretory leucocyte protease inhibitor
TCR: T-cell receptor
TGFβ: transforming growth factor beta
TNFα: Tumor necrosis factor alpha
