# Supplementary figures and images for: Aging beyond menopause selectively decreases CD8+ T cell numbers but enhances cytotoxic activity in the human endometrium
Source: Immun Ageing. 2022 Nov 12;19:55. doi: 10.1186/s12979-022-00312-w (PMC9652910; doi:10.1186/s12979-022-00312-w)

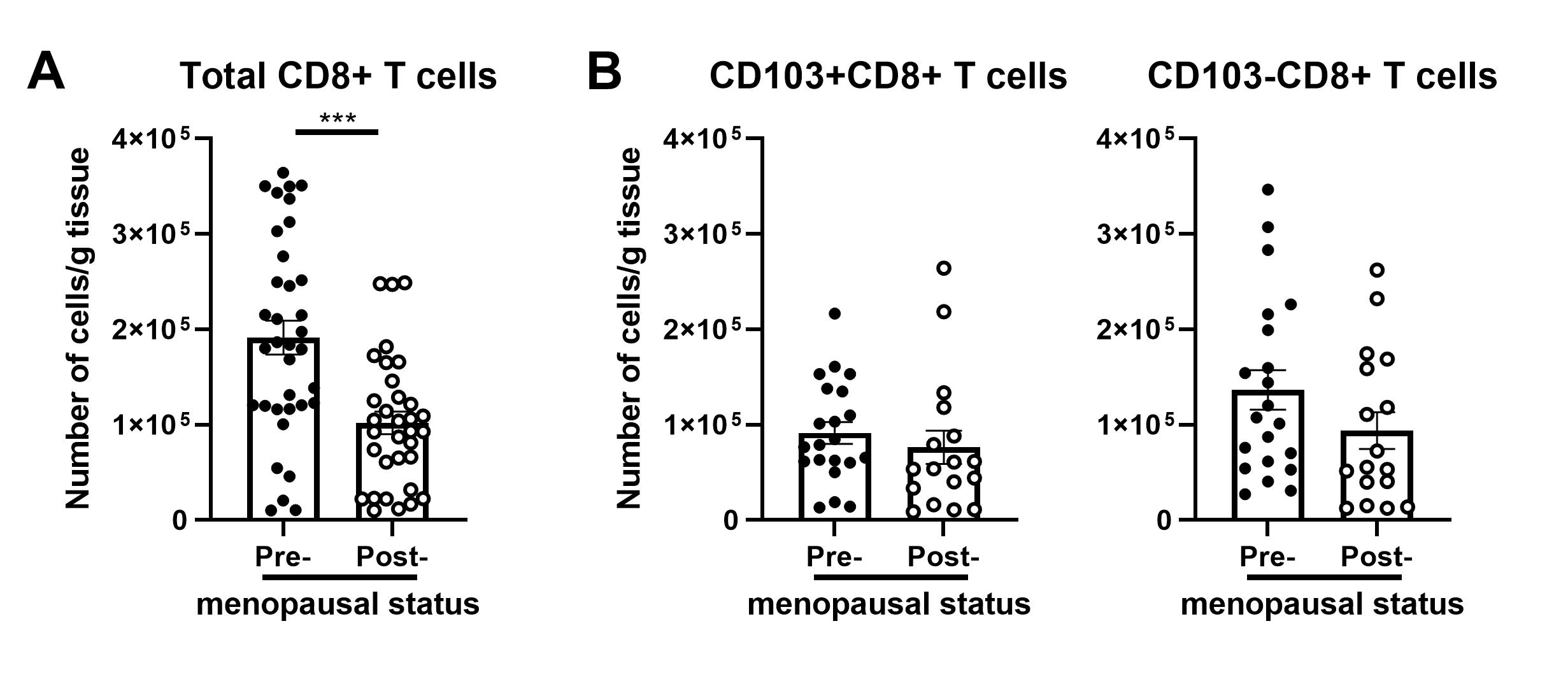

Supplement: Supplementary file 1 — Additional file 1: Supplementary Fig. 1. Menopause differentially regulates CD8+ T cell numbers in EM. (A) Comparison of pre- (black circle; n = 35) vs. post-menopausal (white circle; n = 33) women number of CD8+ T cells recovered per gram of EM tissue after magnetic bead isolation. (B) Comparison of pre- (black circle; n = 21) vs. post-menopausal (white circle; n = 17) women number of CD103+ (left) or CD103- (right) CD8+ T cells recovered per gram of EM tissue after magnetic bead isolation. Each dot represents a single patient. Mean ± SEM are shown. ***P < 0.001; Mann–Whitney U-test. [file 12979_2022_312_MOESM1_ESM.jpg]

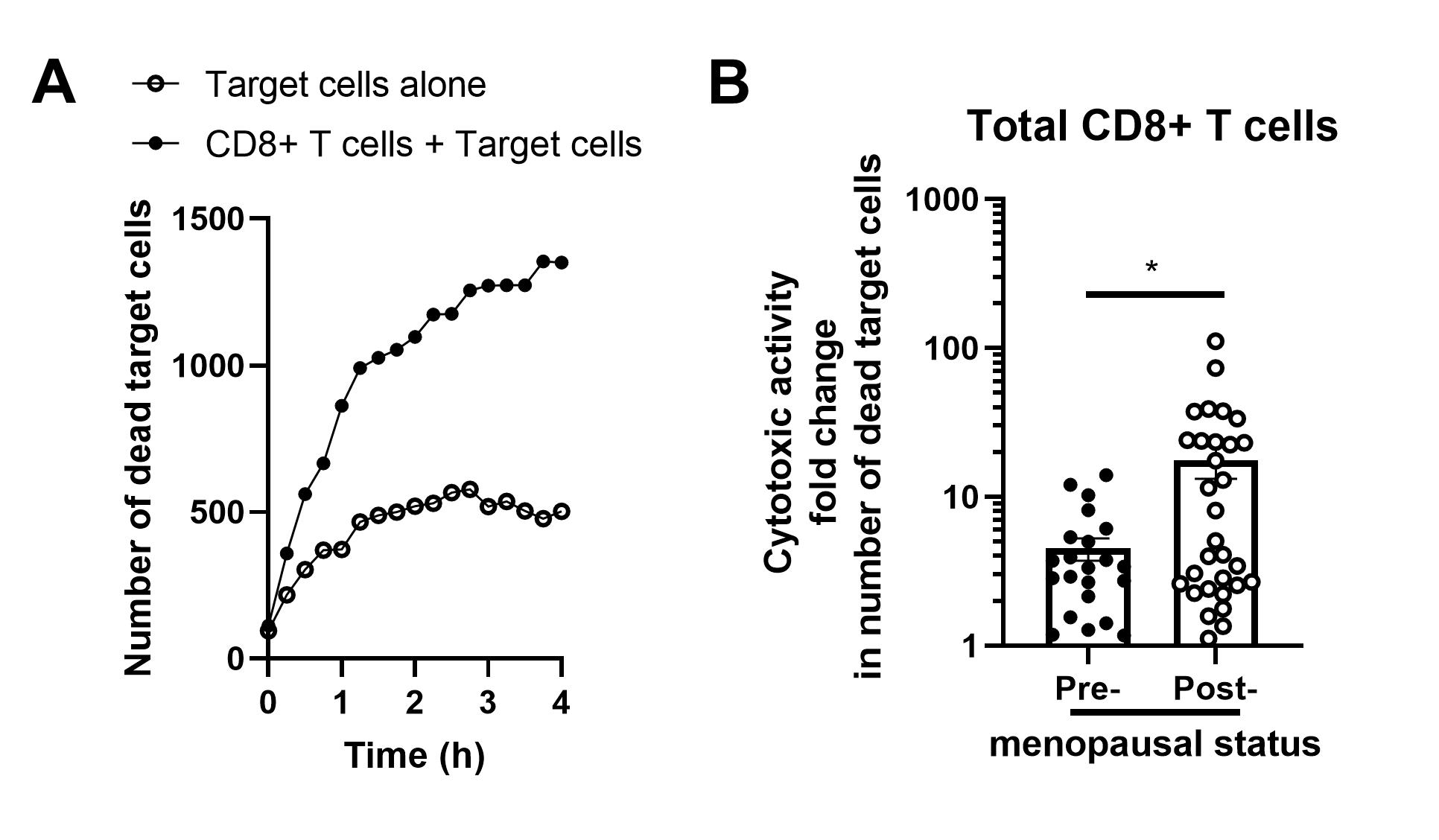

Supplement: Supplementary file 2 — Additional file 2: Supplementary Fig. 2. Menopause differentially regulates CD8+ T cells cytotoxic activity in EM. (A) Representative example of dead target cells kinetics with purified EM CD8+ T cells + target cells (ratio 1:1; dark circle) or target cells alone (open circle) over a period of 4 h. (B) Comparison of pre- (black circle; n = 22) vs. post-menopausal (white circle; n = 31) women EM CD8+ T cells cytotoxic activity. Graph represents the fold change in number of dead target cells in CD8+ T cells + target cells cultures compared to target cell alone. Each dot represents a single patient. Target cells are allogeneic blood CD4+ T cells. Mean ± SEM are shown. *P < 0.05; Mann–Whitney U-test. [file 12979_2022_312_MOESM2_ESM.jpg]

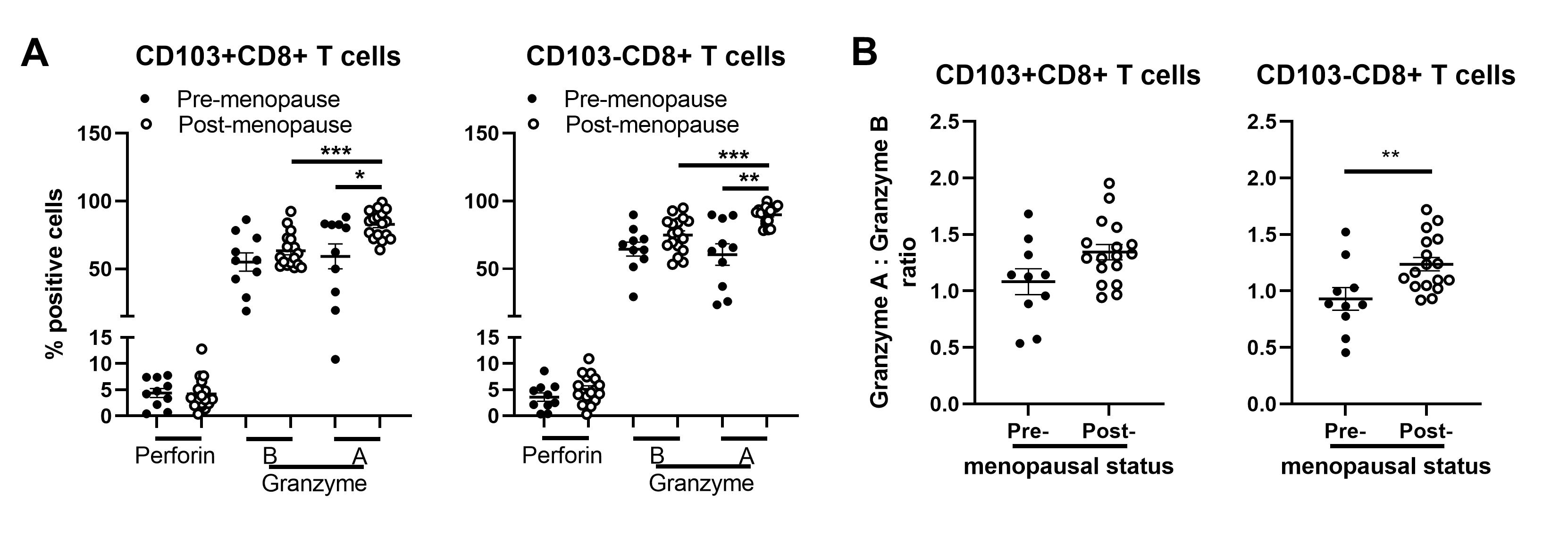

Supplement: Supplementary file 3 — Additional file 3: Supplementary Fig. 3. Menopause differentially regulates CD103+ and CD103-CD8+ T cells intracellular cytotoxic molecules in EM. Mixed cell suspensions from EM tissues were stained for the intracellular cytotoxic molecules perforin, granzyme A and granzyme B for analysis by flow cytometry. (A) Comparison of pre- vs. post-menopausal women percentage positive cells of perforin, granzyme A and granzyme B in EM CD103+ (left) or CD103- (right) CD8+ T cells. (B) Comparison of pre- vs. post-menopausal women the ratio of Granzyme A/Granzyme B from EM CD103+ (left) or CD103- (right) CD8+ T cells. Pre-menopausal women (black circle; n = 10), post-menopausal women (white circle; n = 17). Each dot represents a different patient. Mean ± SEM are shown. *P < 0.05, **P < 0.01, ***P < 0.001; Kruskal-Wallis test followed by Dunns post-test or Friedman test followed by Dunns post-test (A), Mann–Whitney U-test (B). [file 12979_2022_312_MOESM3_ESM.jpg]

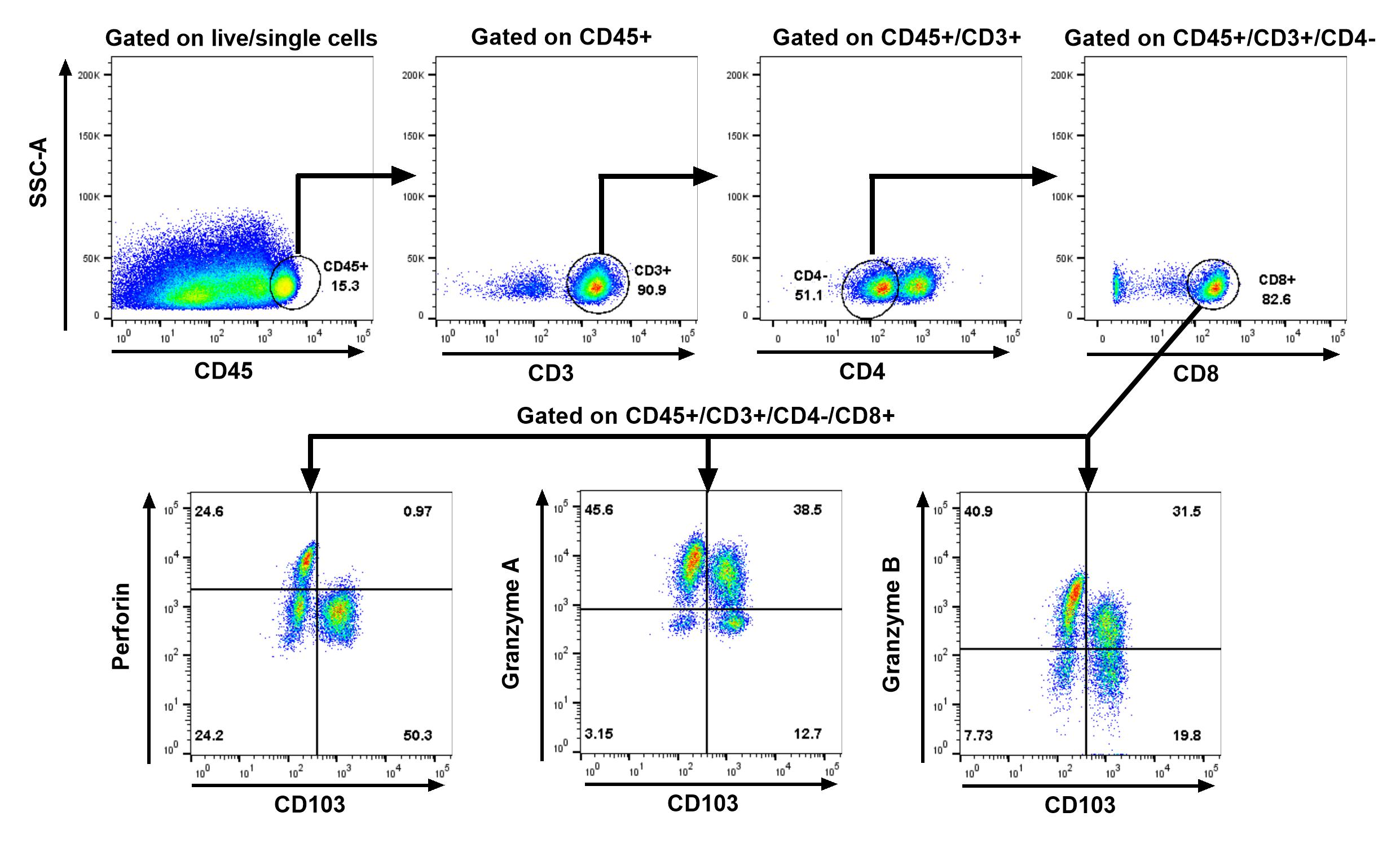

Supplement: Supplementary file 4 — Additional file 4: Supplementary Fig. 4. Gating strategy used for analysis the expression of intracellular perforin, granzyme A and B on CD8+ T cells, or CD103+ and CD103-CD8+ T cells in the mixed cell preparation from endometrium tissue. [file 12979_2022_312_MOESM4_ESM.jpg]
